# A Simple Intervention to Prevent Cutaneous Larva Migrans among Devotees of the Nallur Temple in Jaffna, Sri Lanka

**DOI:** 10.1371/journal.pone.0061816

**Published:** 2013-04-17

**Authors:** Selvam Kannathasan, Arumugam Murugananthan, Nadarajah Rajeshkannan, Nilanthi Renuka de Silva

**Affiliations:** 1 Division of Parasitology, Department of Pathology, Faculty of Medicine, University of Jaffna, Jaffna, Sri Lanka; 2 Department of Community Medicine, Faculty of Medicine, University of Jaffna, Jaffna, Sri Lanka; 3 Department of Parasitology, Faculty of Medicine, University of Kelaniya, Ragama, Sri Lanka; Instituto de Higiene e Medicina Tropical, Portugal

## Abstract

**Background:**

A cross sectional study conducted during the annual festival at Nallur temple in Jaffna, Sri Lanka, in 2010, showed that the prevalence of cutaneous larva migrants (CLM) among the devotees who performed the side roll ritual was 58.2% (95%CI: 51.2%–65.0%).

**Objective:**

To test the hypothesis that the deworming stray dogs around the temple premises effectively reduces the prevalence of CLM among devotees.

**Methodology/Principal Findings:**

All stray dogs (8) in the vicinity of the temple were treated, with mebendazole (100 mg) crushed and filled into sausages, 10 days before the commencement of festival in 2011. The same procedure was repeated a week later to ensure complete coverage. A cross-sectional study was conducted among 200 systematically selected devotees in August 2011 using an interviewer-administered questionnaire and the clinical examination of the skin. Baermann's technique was used for the recovery of nematode larvae from 40 soil samples collected from the temple premises. Ten samples of dog faeces collected from the same premises were also examined for nematode eggs. Prevalence of CLM among devotees in 2010 (Pre intervention) and 2011(Post intervention) were compared to test the hypothesis. Prevalence of CLM declined from 58% to 8% (Chi-square = 112.90, p<0.001) following the intervention. None of the subjects practiced new precautionary measures compared to the previous year. Soil and fecal samples were negative for parasites.

**Conclusions/Significance:**

Regular dog deworming is an important and effective method for the prevention of CLM among the devotees doing side roll ritual and represents a pragmatic intervention that municipal authorities could perform on annual basis.

## Introduction

Cutaneous larva migrans (CLM), also known as creeping eruption [1] of the skin, results from penetration of the human skin by the hookworm larvae of canine, bovine and feline hosts [1], [2]. The typical clinical manifestation of this skin disease is an itchy, erythematous, linear or serpiginous, dermatitis track [1]–[3]. In most instances, the feet, buttocks and thighs [1] represent the main anatomical site affected by CLM, but any part of the body, including trunk, in contact with infested soil or sand can be involved [1], [2].

In 2010, more than half of the studied population (58.2%) of devotees who performed a “side roll” ritual at an annual Hindu festival in Jaffna, Northern Sri Lanka, was found to be affected by this disease [4].

During the festival period, which lasts for 25 days, large numbers of male devotees carry out a ritual known as the ‘side roll’ in fulfillment of vows taken at the temple. In performing this ritual, men lie prostrate on the ground with the upper body uncovered and roll sideways around the temple premises (a distance of approximately 600 m). During the festival period, sand is brought from coastal areas by the Municipal Council and spread around the temple premises in order to reduce the devotees' discomfort. The sand is watered twice daily at about 3.00 am and 5.00 pm in order to keep the dust down.

Though the disease is self-limiting, itching can be intense and may lead to sleep disturbance and secondary infection [5], [6].

The drugs of choice for treatment of CLM in adults are oral ivermectin (single dose of 200 µg/kg body weight) or alternatively oral albendazole (400 mg daily for 5–7 days) [7]–[10]. Even though effective drugs are available, it is very difficult to treat this particular group of patients in Sri Lanka due to their religio-cultural values.

During the festival, strict adherence to daily rituals is the norm. In addition to the side rolling, devotees have a special diet and fasting regimen. Some of them believe that CLM is evidence of the grace of God, with the number of lesions being proportional to the grace. These beliefs, along with the non life threatening nature result in a general lack of concern shown by devotees regarding this condition.

Although contamination of the soil by stray dogs is the most likely source of hookworm [4], the killing of stray dogs is not permitted. Therefore, deworming the stray dogs is the alternative method to prevent the CLM. Hookworm infestation in dogs can be treated with Mebendazole [11].

Therefore, a simple intervention was carried out to test the hypothesis that deworming of stray dogs around the temple premises could reduce the prevalence of CLM among devotees.

## Methods

### Ethics statement

This was the continuation of our previous study [4] for which the ethical clearance was approved by the Ethical Review Committee, Faculty of Medicine, University of Jaffna. Written consent was obtained from the participants. The nature and purpose of the research was explained to the participants, and the information sheet, written in native language (Tamil), was read aloud by the interviewers before obtaining informed written consent. Participants were able to withdraw their consent at any time. Permission to carry out this study was obtained from the commissioner, municipal council, Jaffna and the Nallur Temple Trustee. The devotees identified with CLM lesions referred to Jaffna Teaching Hospital for treatment.

### Study design

As it is more appropriate to conduct this study in a field situation, a quasi-experimental design was chosen for this intervention. One week prior to intervention, the investigators visited the study area three times a day, and calculated the stray dog population. Food parcels were kept in four corners of the temple premises morning, noon and evening, so that all stray dogs that were present in the vicinity could be counted. The total number of dogs observed in the vicinity during the last three days was eight.

When the dogs were used to expecting food, the intervention was carried out 10 days before the commencement of the festival. Mebendazole (100 mg) was crushed, filled into sausages and thrown towards the stray dog. The dogs were observed to ensure the sausages were eaten. The same procedure was repeated to ensure complete coverage.

Two hundred devotees were selected on the last day of the festival in August 2011, using systematic random sampling. Among the first five devotees, the 3^rd^ devotee was selected randomly and thereafter, every 5^th^ devotee was systematically selected until the sample size of 200 was achieved. The annual number of devotees performing the side roll ritual has been steady over the previous three years. Since the sample size was 200 during pre intervention study, similar sample size was maintained (200) to compare the outcome of the intervention.

On the weekend following the festival, the selected devotees were asked to attend the Department of Parasitology in the Faculty of Medicine, Jaffna, for interview and skin inspection. After obtaining written consent, trained medical undergraduates administered a standardized questionnaire in Tamil (the native language of the devotees). The questionnaire covered the demographic details, practice of side roll ritual and characteristic features of skin rash. Moreover, the awareness of CLM and the causative organism, precautionary measures practiced to prevent the disease such as bathing immediately after doing the side roll ritual were also included in the questionnaire. Study participants were also examined by trained medical under graduates and the itchy, erythematous, linear or serpiginous dermatitis lesions were recorded on a pre-set form. Participants with lesions consistent with CLM were confirmed by principal investigator (SK).

In order to identify the source of infection, 20 soil samples (two per truckload) were collected from the sand brought from outside coastal areas before it was spread around the temple premises. A second set of soil samples was collected from the temple premises 10 days after the sand was spread, from 20 sites located every 30^th^ meter along the path where the devotees performed the side roll. Baermann's technique was used for the detection of nematode larvae from the soil samples as described elsewhere [4].

Ten samples of dog faeces collected after the deworming intervention from the same premises were also examined microscopically for the presence of hookworm eggs.

Devotees studied in 2010 and 2011 were considered as pre and post intervention groups respectively (quasi-experimental design). Data were analyzed using statistical package SPSS version 17 and WINPEPI PROGRAMSCOMPARE2 (Version 2.57) J.H. Abramson *Revised April 27, 2011*. Bivariate analysis was done to identify the associated factors. Most of the variables collected as categorical variable. Chi-squared test was used to calculate p values in order to compare the prevalence between two groups. Fishers' exact test was used to compare the proportions of nematode eggs in the faeces and nematode larvae in the soil, before and after intervention.

## Results

All 200 selected devotees accepted the invitation to participate. Among them, the prevalence of CLM was only 8% (95% CI: 1.84–7.45) after this simple intervention (2011), compared to 58.2% among 194 devotees studied in 2010. The statistical difference was extremely significant after deworming (chi-square −112.907, P<0.001). The relative risk reduction of this intervention was 86.3% (95% CI: 77.7–91.5%). The results mirror the good deworming coverage of the stray dogs which significantly reduces the infestation possibilities.

Table 1 compares the background data of the devotees before (2010) and after intervention (2011). Even though some differences noted in age group categories and educational level, both groups are comparable including frequency of potential risk factors for the infestation such as side roll frequency, side roll time, side roll in previous years, symptoms of CLM in previous year, awareness regarding the condition and precautionary measures taken.

**Table 1:** Comparison of study population before and after intervention.

Variable	Categories	Pre Intervention (2010) (n = 194)	Post Intervention (2011) (n = 200)	P value
Age	<19 yrs	23 (12%)	4 (2%)	
	20–40 yrs	146 (75%)	170 (85%)	0.014
	>40 yrs	25 (13%)	26 (13%)	
Education	No formal education	2 (1%)	1(0.5%)	
	Primary	5 (3)%	8(4%)	
	Secondary	157 (81%)	178(89%)	0.029
	Tertiary	30 (15%)	13(6.5%)	
Side roll frequency	Every day	130 (67%)	134 (67%)	0.957
	On selected days	64 (33%)	66 (33%)	
Side roll time	Before 5.00a.m.	112 (58%)	114 (57%)	0.879
	After 5.00a.m	82 (42%)	86 (43%)	
Side roll previous years		173 (89%)	175(87.5%)	0.586
Symptoms of CLM in previous years		77 (40%)	84 (42%)	0.646
Awareness regarding the conditions		157 (81%)	154(77%)	0.356
Any precautionary measures taken		52 (27%)	56(28%)	0.748

Side roll frequency (OR-8.19, 95%CI: 1.06–63.43) and symptoms of CLM in previous year (OR-1.32, 95%CI: 1.12–11.01) were the univariate risk factors for CLM among post intervention group (2011). Multivariate analysis in 2010 (pre intervention) revealed that side roll frequency and the occurrences of a symptoms of CLM in the past were the independent risk factors in 2010.

Table 2 compares the outcome variables before and after intervention. The soil samples examined before and after the spreading around the temple premises were all negative in 2011(after intervention), whereas 2 samples were positive (10%) for nematode larvae in 2010 [4].

**Table 2:** Outcome variables before and after intervention.

Outcomes	Pre Intervention (2010)	Post Intervention (2011)
Prevalence	58.2% (n = 194;95% CI:51.2–65.0)	8% (n = 200;95%CI:1.84–7.45)
Nematode larvae in soil sample	2 samples positive(n = 20)	0 (n = 40) (p = 0.043)
Nematode Eggs in faecal sample	3 samples positive(n = 5)	0 (n = 10) (p = 0.026)

Among the faecal samples collected from the temple premises, three (60%) were positive for hookworm eggs in 2010 (before intervention) whereas all the samples collected after intervention (2011) were negative.

## Discussion

All selected devotees responded to the study (response rate is 100%) and it is more or less same to pre intervention (97%) conducted in 2010 during the Nallur festival season. This small sample size is the major limitation of this study.

Even though the side roll time and awareness regarding the condition were identified as risk factors in univariate analysis among pre intervention group, but not in post intervention group. The possible explanation to this was reduction in the prevalence in 2011 or sampling variation.

A history taken from devotees with CLM revealed it was unlikely they acquired their infection elsewhere and there was no major change in rain fall pattern (Meteorological Department, Jaffna) that might account for differences in CLM prevalence. This suggests that this significant reduction in the prevalence of CLM in 2011 was mainly due to the intervention.

There are few preventive measures that could reduce the incidence of CLM among devotees in festivals like Nallur Temple. The most logical of these would be to secure the temple premises in order to prevent dogs from accessing the grounds and defaecating on the sand. However, this may be difficult to implement, since most of the temple premises in Sri Lanka are not walled off completely at present and stray dogs are numerous.

Reduction in the frequency of watering the sand could reduce soil moisture and thereby, the larval survival. However, this practice adopted for a long time (many years) in Nallur Temple in order to provide the comfort for the devotees. Therefore, this method needs to be balanced against the need to keep down the dust. Further, there was no difference in frequency of watering to the sand and of side roll time between two groups.

Another measure that could prevent infestation is to minimize contact between bare skin and sand by the use of some kind of upper body apparel. However, this may be in conflict with the religious tradition and therefore be unacceptable.

Since the destruction of stray dogs is banned in Sri Lanka by the Government, and due to the religio-cultural believes among the devotees against wearing upper body apparel, deworming the stray dog population is the most possible way to prevent the future epidemics.

Hence, continuing this practice in future, by the authorities (municipal council) responsible for public health activities in the temple and has regular fund allocation in the budget, covering the entire population of strays as much as possible will help to prevent infestation among devotees.

Based on the presented findings, we recommend to continue this practice, by the authorities (municipal council), covering the entire stray dog population as much as possible as an effective method to prevent CLM among devotees in future.

It has to be explored to apply the same strategy to control the CLM in other settings like Brazil [5], [6] and beach destinations such as Jamaica, Barbados, Brazil, Thailand, and Mexico [12] where high prevalence of CLM has been reported.
